# Supplementary material for: The Association of Socioeconomic and Lifestyle Factors with the Oral Health Status in School-Age Children from Pakistan: A Cross-Sectional Study
Source: Healthcare (Basel). 2023 Mar 4;11(5):756. doi: 10.3390/healthcare11050756 (PMC10001539; doi:10.3390/healthcare11050756)
Supplement: Supplementary file 1 [file healthcare-11-00756-s001.zip › healthcare-2117435-supplementary.pdf]

Supplementary Table S1: Post hoc analysis of dependent and independent variables in this study (n=359)

Dependent variables	Comparisons	Adjusted *p* value	*p*-value
**Gender (Male, Female)**	Male vs BT < 1 minute	0.36812	0.006*
	Male vs BT 2-3 minutes	0.68916	
	Male vs BT > 2-3 minutes	0.92034	
	Male vs BT <2-3 minutes	0.61708	
	Female vs BT < 1 minute	0.36812	
	Female vs BT 2-3 minutes	0.68916	
	Female vs BT > 2-3 minutes	0.92034	
	Female vs BT < 2-3 minutes	0.61708	
	Male vs BF once a day	0.00067*	0.008*
	Male vs BF twice a day	0.00067*	
	Male vs BF thrice a day	0.84148	
	Female vs BF once a day	0.00067*	
	Female vs BF twice a day	0.00067*	
	Female vs BF thrice a day	0.84148	
**Level of maternal education (Educated, Un educated)**	Edu vs BT < 1 minute	0.31731	0.006*
	Edu vs BT 2-3 minutes	0.10960	
	Edu vs BT > 2-3 minutes	0.00097	
	Edu vs BT < 2-3 minutes	0.16151	
	Un Edu vs BT < 1 minute	0.31731	
	Un Edu vs BT 2-3 minutes	0.10960	
	Un Edu vs BT > 2-3 minutes	0.00097*	
	Un Edu vs BT < 2-3 minutes	0.16151	
**Physical Activity (everyday, once a week, twice a week, rarely, never)**	Everyday vs PSP	0.36812	0.005*
	Everyday vs PSA	0.36812	
	OW vs PSP	0.92034	
	OW vs PSA	0.92034	
	TW vs PSP	0.76418	
	TW vs PSA	0.76418	
	Rarely vs PSP	0.19360	
	Rarely vs PSA	0.19360	
	Never vs PSP	0.01640	
	Never vs PSA	0.01640	

**Physical Activity (everyday, once a week, twice a week, rarely, never)**	*Everyday vs Sugary Drink*	0.27133	0.003*
	Everyday vs Non-Sugary Drink	0.23014	
	Everyday vs Both	0.61708	
	OW vs Sugary Drink	0.36812	
	OW vs Non-Sugary Drink	0.48393	
	OW vs Both	0.42371	
	TW vs *Sugary Drink*	0.13361	
	TW vs Non-Sugary Drink	0.10960	
	TW vs Both	0.61708	
	Rare vs Sugary Drink	0.68916	
	Rare vs *Non-Sugary Drink*	0.36812	
	Rare vs Both	0.00137*	
	Never vs *Sugary Drink*	0.13361	
	Never vs *Non-Sugary Drink*	0.19360	
	Never vs Both	0.42371	
**Physical Activity vs Absenteeism (everyday, once a week, twice a week, rarely, never)**	*Everyday vs yes*	0.00373*	0.005*
	Everyday vs Never	0.00373*	
	OW vs Yes	0.16151	
	OW vs Never	0.16151	
	TW vs Yes	0.07186	
	TW vs Never	0.07186	
	Rarely vs Yes	0.02145	
	Rarely vs Never	0.48393	
	Never vs yes	0.48393	
	Never vs Never	0.54851	
**Self-care oral health (good, average, poor)**	Good vs Yes	0.08913	0.008*
	Good vs Never	0.00693*	
	Average vs Yes	0.00693*	
	Average vs Never	0.07186	
	Poor vs Yes	0.07186	
	Poor vs Never	0.84148	
**Grades (Class I,II, III, IV, V, VI, VII, VIII, IX)**	*Class I vs BF once a day*	0.23014	0.001*
	Class I vs BF twice a day	0.27133	
	Class I vs BF thrice a day	0.31731	
	Class II vs BF once a day	0.23014	
	Class II vs BF twice a day	0.31731	
	Class II vs BF thrice a day	0.54851	
	Class III vs BF once a day	0.48393	

	Class III vs BF twice a day	0.42371	
	Class III vs BF thrice a day	0.76418	
	Class IV vs BF once a day	0.00511*	
	Class IV vs BF twice a day	0.01242	
	Class IV vs BF thrice a day	0.03573	
	Class V vs BF once a day	0.84148	
	Class V vs BF twice a day	0.84148	
	Class V vs BF thrice a day	0.68916	
	Class VI vs BF once a day	0.36812	
	Class VI vs BF twice a day	0.31731	
	Class VI vs BF thrice a day	0.84148	
	Class VII vs BF once a day	0.01640	
	Class VII vs BF twice a day	0.01640	
	Class VII vs BF thrice a day	0.76418	
	Class VIII vs BF once a day	0.36812	
	Class VIII vs BF twice a day	0.36812	
	Class VIII vs BF thrice a day	0.92034	
	Class IX vs BF once a day	0.54851	
	Class IX vs BF twice a day	0.48393	
	Class IX vs BF thrice a day	0.42371	

**Significant p-value, BT=brushing time, Edu=educated, Un edu= uneducated, PSP=physical activity present, PSA= physical activity absent, OW=once a day, TW=twice a day*
